# The expression of MDM2 in gastrointestinal stromal tumors: immunohistochemical analysis of 35 cases

**DOI:** 10.1186/s12907-018-0069-0

**Published:** 2018-01-24

**Authors:** Boubacar Efared, Gabrielle Atsame-Ebang, Layla Tahiri, Ibrahim Sory Sidibé, Fatimazahra Erregad, Nawal Hammas, Samia Arifi, Ihsane Mellouki, Abdelmalek Ousadden, Khalid Mazaz, Hinde El Fatemi, Laila Chbani

**Affiliations:** 1 Department of pathology, Hassan II university hospital, Fès, Morocco; 2 Laboratory of biological and translational research, Faculty of pharmacology and medicine, Sidi Mohamed Ben Abdellah University, Fès, Morocco; 3 Department of medical oncology, Hassan II university hospital, Fès, Morocco; 4 Faculty of pharmacology and medicine, Sidi Mohamed Ben Abdellah University, Fès, Morocco; 5 Department of hepatogastroenterology, Hassan II university hospital, Fès, Morocco; 6 Department of general and visceral surgery, Hassan II university hospital, Fès, Morocco

**Keywords:** Gastrointestinal stromal tumors (GIST), MDM2, Immunohistochemistry, Histoprognosis

## Abstract

**Background:**

Gastrointestinal stromal tumors (GIST) are the most common primary mesenchymal tumors of the digestive system. The assessment of their biological behavior still remains a scientific challenge. To date, there are no well-established biological prognostic markers of GIST. Our aim is to study the expression of the MDM2 oncoprotein in GIST through an immunohistochemical analysis.

**Methods:**

It was a retrospective study of 35 cases of GIST diagnosed from 2009 to 2012 in the department of pathology of Hassan II university hospital, Fès, Morocco. MDM2 immunohistochemical staining was performed on archival paraffin-embedded and formalin-fixed specimens (with a threshold of nuclear positivity > 10%). Analysis of correlations between MDM2 immunoexpression and clinicopathological features of GIST has been performed.

**Results:**

The mean age was 55.23 years (range 25–84 years) with a male predominance (sex ratio = 1.5). The stomach was the main site of GIST, with 17 cases (48.57%) followed by the small bowel (9 cases, 25.71%). The spindle cell type GIST was the most frequent morphological variant (29 cases, 82.85%). Tumor necrosis was present in 8 cases (22.85%). Two patients (5.71%) had very low risk GIST, 5 (14.28%) had low risk GIST, 7 patients (20%) had intermediate risk tumors. The remaining 21 cases (60%) had high risk GIST. At the time of diagnosis, 9 patients (25.71%) had metastatic tumors. At immunohistochemical analysis, 40% of cases (14 patients) stained positive for MDM2. Of these MDMD2-positive tumors, 11/14 (78.57%) had high risk tumors and 8/14 cases (57.14%) presented with metastatic GIST. MDM2 positivity was significantly associated with the metastatic status (*p* = 0.001).

**Conclusion:**

The current study suggests that MDM2 immunohistochemical expression is a negative histoprognostic factor in GIST with a statistically significant correlation with metastasis.

## Background

Gastrointestinal stromal tumors (GIST) are the most common primary mesenchymal tumors of the digestive system [1, 2]. They constitute a wide spectrum of neoplasms with characteristic histological, immunohistochemical and molecular features. The most common genetic alterations found in GIST include mutations of growth factors genes such as *KIT* (70–80%) and *PDGFRA* (platelet-derived growth factor A) (5–15%) [2–6]. To date, much is known about the histological, immunohistochemical and molecular aspects of GIST especially in diagnostic purposes, it is however obvious that little is known about the clinicopathological features that can predict the biological behavior of these tumors. In fact, several features of GIST have been postulated in the past to predict their clinical behavior [1, 7–10]. The widely accepted risk stratification of GIST is known as AFIP (Armed Forces Institute of Pathology) criteria, reported by Miettinen et al. This system of risk stratification is in fact a modification of a NIH (National Institutes of Health) consensus criteria [1, 9, 10]. To determine the risk of recurrence, the AFIP criteria takes into account tumor size and mitotic count/50 HPF (high power field), according to the anatomic location of the tumor. Thus, GIST are subdivided into very low, low, intermediate and high risk tumors [1]. Beside these systems of risk stratification, several attempts have been made to identify molecules or genetic alterations that can have a prognostic value in determining GIST behavior [11–13]. As a mesenchymal tumor, alterations of oncogenes (or their products) like *MDM2* (Murine Double Minute 2) or *TP53*, have been widely investigated through various techniques in GIST [11, 12]. In a similar perspective, herein we have tried to study the immunohistochemical overexpression of MDM2 in GIST, and its correlations with other clinicopathological parameters.

## Methods

A part of this study has been presented as an E-poster (E-PS-06-036: MDM2 as a prognostic marker for GIST: A retrospective study of 43 cases) at the 28th European Congress of Pathology and published as an abstract (Virchows Arch (2016) 469 (Suppl 1):S1–S346).

### Patients selection

**Fig. 1:**
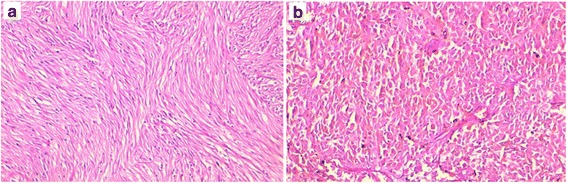
The histomorphological types of gastrointestinal stromal tumors (GIST) in our study. **a** A spindle cells type with fusocellular cells disposed in intersected fascicles. **b** The epithelioid GIST variant shows a solid architecture with cohesive polygonal cells and oval nucleis. (HES × 200)

**Fig. 2:**
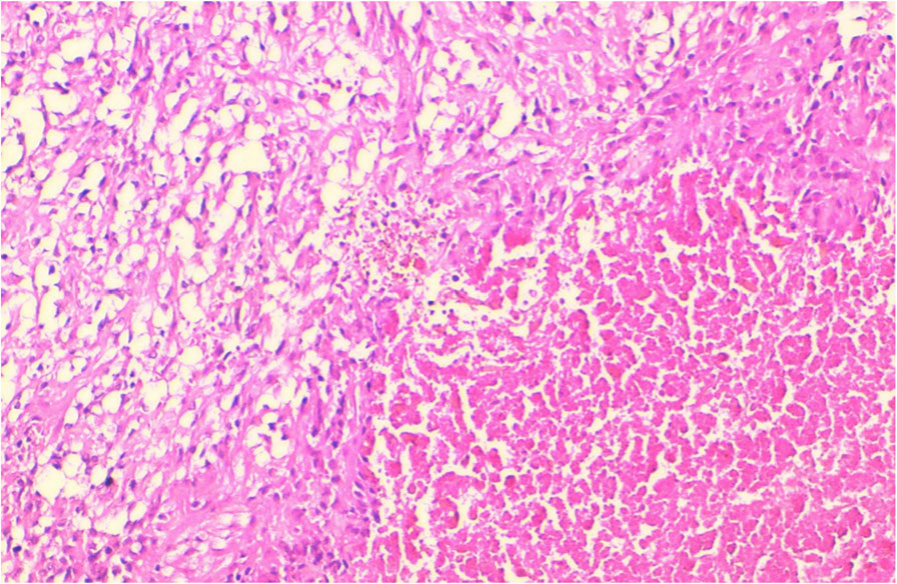
The histological view of a GIST tumor with prominent necrosis (HES × 200)

**Fig. 3:**
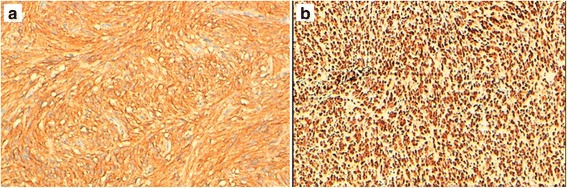
**a** Example of a diffuse cytoplasmic immunoexpression of CD117 by a case of GIST in our study. **b** A case of GIST showing intense nuclear staining with anti-MDM2 antibody. (× 200)

The histological sections have been retrospectively retrieved from 35 patients diagnosed with gastrointestinal stromal tumors (GIST) from 2009 to 2012 in the department of pathology of Hassan II University hospital, Fès, Morocco. Clinical and histopathological data have been recorded from pathology requests forms and the patients’ medical records. The initial diagnosis of GIST has been made on paraffin-embedded and formalin-fixed specimens after staining with hematoxylin-eosine-safran (HES) (Figs. 1a, b and 2). In all cases, the diagnosis of GIST has been retained after immunohistochemical analysis that showed unequivocal diffuse and intense membranous or cytoplasmic expression of CD117 (Fig. 3a), and after excluding potential differential diagnosis by using commonly utilized panel of antibodies such as anti-CD34, anti-S-100 protein and anti-SMA (smooth muscle actin) [1–4, 6]. The risk assessment of GIST has been based on the AFIP criteria [1].

### Immunohistochemistry

MDM2 immunohistochemical staining was performed on archival paraffin-embedded and formalin-fixed specimens from all the 35 patients previously diagnosed with GIST. We have used the anti-MDM2 antibody according to the manufacturer’s guidelines, with an automated immunohistochemical stainer (Ventana BenchMark ULTRA®). A threshold of nuclear positivity > 10% has been fixed as a positive MDM2 staining (Fig. 3b).

### Statistics

The statistical analysis was performed by using SPSS® 20.0. The chi-square test or the Fisher exact test when appropriate, have been used to assess correlations between MDM2 immunoexpression with different features of GIST (risk assessment, metastatic status, tumor site, size, tumor necrosis and mitotic count). Results were statistically significant when *p ˂ 0.05*.

## Results

**Fig. 4:**
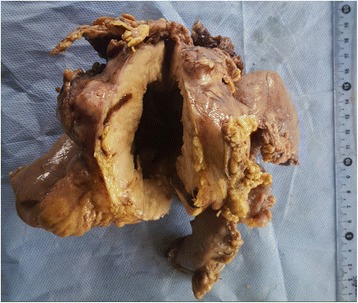
A resected specimen of a small bowel GIST. The tumor arises typically in the intestinal wall and presents a cystic cavitation

Table 1 summarizes the clinicopathological features of our 35 patients. The diagnosis of GIST has been made on 13 biopsies and on 22 surgical resected specimens (Fig. 4). The mean age was 55.23 years (range of 25-84 years); there was a slight male predominance, with 21 male patients and 14 women (sex ratio = 1.5). The stomach was the main site of GIST, with 17 cases (48.57%), followed by the small bowel with 9 cases (25.71%) (Fig. 4) and the peritoneum (5 cases, 14.28%). The duodenum and the colon were rarely affected, respectively in 8.57% (3 patients) and 2.85% (1 patient). The spindle cell type (Fig. 1a) was the most frequent histological variant of GIST in our study, with 29 cases (82.85%) while the epithelioid variant was found in only one patient (Fig. 1b). The mixed variant, spindle and epithelioid cell type was seen in 5 cases (14.28%). Tumor necrosis was present in 8 cases (22.85%).

**Table 1:** Clinicopathological features of our 35 patients diagnosed with GIST

Cases	Age (year)	Sex	Site	Histol type	Necrosis	Risk	Metastatic site	MDM2
1	40	F	Stomach	Sp cel	–	High	Peritoneum	+
2	53	M	Stomach	Sp cel	–	High	Peritoneum, Liver	+
3	65	F	Peritoneum	Sp + Ep cel	+	High	Liver, lung	+
4	42	M	Small bowel	Sp cel	–	Low	–	–
5	40	F	Duodenum	Sp cel	+	Intermediate	Peritoneum	+
6	50	F	Small bowel	Sp cel	–	Low	–	+
7	35	M	Small bowel	Sp cel	–	Intermediate	–	–
8	84	M	Small bowel	Sp cel	+	High	Liver	+
9	60	M	Peritoneum	Sp cel	–	High	–	+
10	50	M	Duodenum	Sp cel	–	Intermediate	–	+
11	43	F	Stomach	Sp cel	+	High	–	–
12	56	M	Small bowel	Sp + Ep cel	+	High	Peritoneum	+
13	64	F	Colon	Sp cel	–	Low	–	–
14	54	F	Stomach	Ep cel	+	High	–	–
15	72	M	Stomach	Sp cel	–	High	Peritoneum, liver, lung, adrenal gland	+
16	60	F	Stomach	Sp cel	–	High	–	+
17	52	M	Small bowel	Sp cel	–	High	–	+
18	70	M	Stomach	Sp cel	–	Intermediate	–	+
19	55	M	Small bowel	Sp cel	–	High	–	–
20	50	M	Stomach	Sp + Ep cel	–	High	Peritoneum	+
21	50	M	Stomach	Sp cel	–	Very low	–	–
22	48	M	Duodenum	Sp cel	–	Low	–	–
23	45	M	Stomach	Sp + Ep cel	+	High	–	+
24	83	M	Stomach	Sp cel	–	High	–	–
25	70	M	Small bowel	Sp cel	–	High	Lung, liver	–
26	25	F	Small bowel	Sp cel	–	Low	–	–
27	70	F	Stomach	Sp + Ep cel	–	Intermediate	–	–
28	49	M	Stomach	Sp cel	–	Intermediate	–	–
29	58	F	Peritoneum	Sp cel	–	High	–	–
30	52	M	Stomach	Sp cel	–	Intermediate	–	–
31	64	M	Stomach	Sp cel	–	High	–	–
32	56	M	Peritoneum	Sp cel	–	High	–	–
33	30	F	Peritoneum	Sp cel	+	High	–	–
34	57	F	Stomach	Sp cel	–	Very low	–	–
35	37	F	Stomach	Sp cel	–	High	–	–

*F* female, *M* male, *Sp cel* spindle cells type, *Sp + Ep cel* spindle and epithelioid type, *Ep* epithelioid type, − absent, negative, + present, positive

According to the AFIP criteria of the risk assessment, 2 patients (5.71%) had very low risk GIST, 5 (14.28%) had low risk GIST, 7 patients (20%) had intermediate risk tumors. The remaining 21 cases (60%) had high risk GIST. At the time of diagnosis, 9 patients presented with synchronous metastasis. The peritoneal metastasis were the most frequent (4 cases, 11.42%). Other organs like the liver, the lung and the adrenal gland, have been involved by metastatic tumors (Table 1).

The immunohistochemical analysis showed 14 cases positive for MDM2 (40%) (Fig. 3b).

Among the 9 metastatic GIST, 8 had MDM2-positive tumors, showing a statistical significant correlation of MDM2 positivity with metastatic status (*p* = 0.001). Also, our current study showed that MDM2-positive GIST had a tendency to have a higher size (> 10 cm) as well as a higher mitotic count (> 5 mitosis/50 HPF) (*p* = 0.08 and 0.06 respectively) (Table 2).

**Table 2:** Correlation of MDM2 immunostaining with tumor site, size, mitotic count, necrosis, tumor risk, and metastastatic status

Variables	MDM2 negative (%)	MDM2 positive (%)	*P* value
Tumor site:			
Stomach	11/17 (64.70%)	6/17 (35.29%)	0.87
S. bowel	5/9 (55.55%)	4/9 (44.44%)	
Peritoneum	3/5 (60%)	2/5 (40%)	
Duodenum	1/3 (33.33%)	2/3 (66.66%)	
Colon	1/1 (100%)	0/1 (0%)	
Size (cm):			
≤ 5	7/8 (87.5%)	1/8 (12.5%)	0.08
5˂ S ≤ 10	7/17 (41.17%)	10/17 (58.82%)	
> 10	7/10 (70%)	3/10 (30%)	
Mitotic count/50 HPF:			
≤ 5	11/14 (78.57%)	3/14 (21.42%)	0.06
> 5	10/21 (47.61%)	11/21 (52.38%)	
Necrosis:			
Absent	18/27 (66.66%)	9/27 (33.33%)	0.14
Present	3/8 (37.5%)	5/8 (62.5%)	
Risk:			
Very low	2/2 (100%)	0/2 (0%)	0.34
Low	4/5 (80%)	1/5 (20%)	
Intermediate	5/7 (71.42%)	2/7 (28.57%)	
High	10/21 (47.61%)	11/21 (52.38%)	
Metastasis:			
Absent	20/26 (76.92%)	6/26 (23.07%)	0.001
Present	1/9 (11.11%)	8/9 (88.88%)	

## Discussion

The epidemiological and histological characteristics of our current study are approximately similar to what has been previously reported [1–4]. The mean age was 55.23 years with a slight male predominance (21 men for 14 women). All of our patients were adults, with age ranging from 25 to 84 years. Gastrointestinal stromal tumors (GIST) are usually encountered in middle-aged or elderly adults, affecting very rarely the pediatric population [3]. Unlike in adults where GIST usually affect equally both sex, in young patients the female predominance is common and tumors present as a component of cancer predisposing syndroms (Carney triad and Carney-Stratakis syndrom) [1, 3, 14]. The stomach is the most site of GIST followed by the small intestine, other parts of the gastrointestinal tract such as the colon or the esophagus are rarely affected [2, 4]. However, a small subset of GIST has been found to occur in extraintestinal wall, generally in the vicinity of the gastrointestinal tract (GI tract), especially in the omentum, the mesentery, or the retroperitoneum. These tumors are termed as extragastrointestinal stromal tumors (EGIST) and have histological and genetic features similar to those of the common GIST [3, 15, 16]. We have found 5 cases of peritoneal EGIST among our 35 patients. The vast majority of GIST were located in the stomach (17 cases, 48.57%) and in the small bowel (9 cases, 25.71%).

The histological features of our patients were consistent with those previously reported in the literature [1, 3]. The spindle cells variant is the most frequent histological subtype of GIST, followed by the epithelioid variant. We have recorded 29 cases (82.85%) of spindle cells GIST, 1 case of epithelioid variant and 5 mixed-subtypes (14.28%) showing admixture of epithelioid and spindle tumoral cells. We have not recorded rare morphologic variants like the sclerosing epithelioid or spindle cells subtype, the sarcomatoid variants or the palisading vacuolated spindle cell subtype. In our study, tumor necrosis was found in 22.45% (8 cases), and approximately 22–37% of GIST are associated with necrosis as reported in the literature [1]. On immunohistochemistry, GIST stain positive for CD117 (95%), with a small subset (5%) that can be negative for this marker. DOG1 is almost constantly expressed by GIST regardless of the mutational status [2–4]. Muscle markers and the S-100 protein can be weakly expressed by GIST, the CD34 immunoexpression is frequent in GIST, varying around 50–100% [1, 6]. In fact, these markers are not a “gold standard” for the positive diagnosis of GIST but can prove useful in order to rule out potential differential diagnosis. The most valuable markers are CD117 and DOG1 for the positive diagnosis [17]. In our study, for the diagnostic purpose, we have used an immunohistochemical panel comprising antibodies against CD117, CD34, SMA and S-100 protein. All of our cases (100%) showed a diffuse and strong membranous or cytoplasmic staining for CD117, 27 cases (77.14%) were positive for CD34, 6 cases (17.14%) had a weak staining for SMA, whereas 1 case was weakly positive for S-100. The fact that we have not used DOG1 at the time of our study has certainly limited the chance of recording CD117-negative GIST, especially the epithelioid subtype that can be CD117-negative in certain cases with *PDGRA* mutations or in other genetic alterations [2].

Like many cancers, the prognosis of GIST is based upon the occurrence of metastasis. In the past, a number of systems have been designed to assess the risk of recurrence or metastasis occurence in GIST [1, 7–10]. The AFIP criteria, reported by Miettienen et al. is widely used to assess the prognosis of GIST [1]. According to this system, 60% of patients (21 cases) in our study had high risk tumors, 7 patients (20%) had intermediate risk GIST, while the remaining cases had low risk and very low risk tumors (14.28% and 5.71% respectively). As synchronous metastasis have been diagnosed in 25.71% of our patients (9 cases), we have tried to correlate this clinical aggressive behavior with MDM2 immunoexpression. Although the AFIP criteria remains the most widely accepted model of predicting GIST behavior, other systems of risk stratification have been proposed, especially by Joensuu et al. These authors have suggested to consider the tumor rupture as a prognostic criteria along with the tumor site and the mitotic count [7, 9].

Concurrently, many attempts have been made to find prognostic biological markers to predict the behavior of GIST [11–13]. As a stromal tumor with a potential risk of malignancy (high risk tumors), GIST have been expected to harbor molecular or genetic disorders commonly found in various sarcomas. One of the most investigated molecular aspect in GIST is the MDM2-p53 pathway [11, 12, 18, 19]. *MDM2* is amplified in many human sarcomas, and at least 50% of human cancers harbor *TP53* mutations [19, 20]. In fact, *MDM2* is an oncogene that mainly exerts its activity by downregulating the *TP53* tumor suppressor gene activity and its product, p53. MDM2 negatively regulates p53 through its E3 ubiquitin ligase property. In fact, MDM2 binds to p53 and leads to its proteasomal degradation [18, 19, 21]. In 2005, Tornillo et al. found that around 10% of high risk/malignant GIST showed amplification of *MDM2* oncogene, and concluded that this fact may have a prognostic relevance in GIST [11]. However, more recently Wallander et al. found that amplification of *MDM2* is uncommon in GIST and it did not correlate with the tumoral behavior [12]. These studies have focused on *MDM2* amplification by using fluorescent in situ hybridization (FISH) analysis. In fact, MDM2 oncoprotein overexpression can be a result of either its gene mutation or a consequence of other post-transcriptional regulatory mechanisms [18, 19]. The proteomic approach is a best indicator of overexpression of oncoproteins like MDM2, regardless of biological mechanisms underlying their overproduction. Unfortunately this approach has been rarely applied to GIST [22]. We thought that immunohistochemistry, by showing overexpression of a given antigen, reflects partially the proteomic approach. In our current study, we have tried to assess overexpression of the MDM2 oncoprotein and its correlations with clinicopathological features of GIST. We found that 40% (14 cases) of GIST has shown immunohistochemical overexpression of MDM2, with 11/14 cases harboring high risk tumors and 8/14 cases presented with metastatic tumors. MDM2 immunohistochemical overexpression has been significantly associated with the metastatic status (*p* = 0.001). Despite the small size of our sample, we suggest that MDM2 immunohistochemical expression may have a prognostic significance in GIST, and this fact emphasizes the need for large studies to show the exact prognostic value of MDM2 oncoprotein. Recent studies have shown a great therapeutic promise of pharmacologic agents that modulate the MDM2-p53 pathway in GIST and in other types of cancers [23–25]. Interestingly, a new favorable prognostic biomarker of GIST, named pfetin has been discovered and its immunohistochemical assessment has proven useful in predicting recurrences or metastasis in GIST [22, 26]. The next years will probably provide significant insights about the identification of relevant prognostic biomarkers of GIST through robust scientific evidences.

## Conclusion

Gastrointestinal stromal tumors (GIST) are common mesenchymal tumors of the gastrointestinal tract, with characteristic histopathological features. However the assessment of their biological behavior still remains a significant challenge. The current study suggests that MDM2 immunohistochemical expression is a negative histoprognostic factor in GIST and is significantly associated with the metastatic risk. These findings emphasize the urgent need for large studies in this way as the therapeutic modulation of MDM2-p53 pathway shows a consistent promise.

## Abbreviations

AFIP: Armed Forces Institute of Pathology
GIST: Gastrointestinal stromal tumors
HES: Hematoxylin-eosin-safran
MDM2: Murine double minute 2
PDGFRA: Platelet-derived growth factor receptor A

## References

[1] Miettinen M, Lasota J (2006). Gastrointestinal stromal tumors: pathology and prognosis at different sites. Semin Diagn Pathol.

[2] Rubin BP, Heinrich MC (2015). Genotyping and immunohistochemistry of gastrointestinal stromal tumors: an update. Semin Diagn Pathol.

[3] Yamamoto H, Oda Y (2015). Gastrointestinal stromal tumor: recent advances in pathology and genetics. Pathol Int.

[4] Lamba G, Gupta R, Lee B, Ambrale S, Liu D (2012). Current management and prognostic features for gastrointestinal stromal tumor (GIST). Exp Hematol Oncol.

[5] Nannini M, Astolfi A, Urbini M, Indio V, Santini D, Heinrich MC (2014). Integrated genomic study of quadruple-WT GIST (KIT/PDGFRA/SDH/RAS pathway wild-type GIST). BMC Cancer.

[6] Shidham VB, Chivukula M, Gupta D, Rao RN, Komorowski R (2002). Immunohistochemical comparison of gastrointestinal stromal tumor and solitary fibrous tumor. Arch Pathol Lab Med.

[7] Joensuu H (2008). Risk stratification of patients diagnosed with gastrointestinal stromal tumor. Hum Pathol.

[8] Gronchi A (2013). Risk stratification models and mutational analysis: keys to optimising adjuvant therapy in patients with gastrointestinal stromal tumour. Eur J Cancer.

[9] Joensuu H, Vehtari A, Riihimäki J, Nishida T, Steigen SE, Brabec P (2012). Risk of recurrence of gastrointestinal stromal tumour after surgery: an analysis of pooled population-based cohorts. Lancet Oncol.

[10] Racz JM, Brar SS, Cleghorn MC, Jimenez MC, Azin A, Atenafu EG (2015). The accuracy of three predictive models in the evaluation of recurrence rates for gastrointestinal stromal tumors. J Surg Oncol.

[11] Tornillo L, Duchini G, Carafa V, Lugli A, Dirnhofer S, Di Vizio D (2005). Patterns of gene amplification in gastrointestinal stromal tumors (GIST). Lab Investig.

[12] Wallander ML, Layfield LJ, Tripp SR, Schmidt RL (2013). Gastrointestinal stromal tumors: clinical significance of p53 expression, MDM2 amplification, and KIT mutation status. Appl Immunohistochem Mol Morphol.

[13] Dorn J, Spatz H, Schmieder M, Barth TF, Blatz A, Henne-Bruns D (2010). Cyclin H expression is increased in GIST with very-high risk of malignancy. BMC Cancer.

[14] Janeway KA, Weldon CB (2012). Pediatric gastrointestinal stromal tumor. Semin Pediatr Surg.

[15] Arabi NA, Musaad AM, Ahmed EE, Abdo AA, Elhassan AM, Hassan H (2014). Primary extragastrointestinal stromal tumour of the whole abdominal cavity, omentum, peritoneum and mesentery: a case report and review of the literature. J Med Case Rep.

[16] Saeed Z, Taleb S, Evans-Molina C (2016). A case of extragastrointestinal stromal tumor complicated by severe hypoglycemia: a unique presentation of a rare tumor. BMC Cancer.

[17] Novelli M, Rossi S, Rodriguez-Justo M, Taniere P, Seddon B, Toffolatti L (2010). DOG1 and CD117 are the antibodies of choice in the diagnosis of gastrointestinal stromaltumours. Histopathology.

[18] Zhao Y, Yu H, Hu W (2014). The regulation of MDM2 oncogene and its impact on human cancers. Acta Biochim Biophys Sin Shanghai.

[19] Wade M, Li YC, Wahl GM (2013). MDM2, MDMX and p53 in oncogenesis and cancer therapy. Nat Rev Cancer.

[20] Flørenes VA, Maelandsmo GM, Forus A, Andreassen A, Myklebost O, Fodstad O (1994). MDM2 gene amplification and transcript levels in human sarcomas: relationship to TP53 gene status. J Natl Cancer Inst.

[21] Inoue K, Fry EA, Frazier DP (2016). Transcription factors that interact with p53 and Mdm2. Int J Cancer.

[22] Kondo T, Suehara Y, Kikuta K, Kubota D, Tajima T, Mukaihara K (2013). Proteomic approach toward personalized sarcoma treatment: lessons from prognostic biomarker discovery in gastrointestinal stromal tumor. Proteomics Clin Appl.

[23] Henze J, Mühlenberg T, Simon S, Grabellus F, Rubin B, Taeger G (2012). p53 modulation as a therapeutic strategy in gastrointestinal stromal tumors. PLoS One.

[24] Zanjirband M, Edmondson RJ, Lunec J (2016). Pre-clinical efficacy and synergistic potential of the MDM2-p53 antagonists, Nutlin-3 and RG7388, as single agents and in combined treatment with cisplatin in ovarian cancer. Oncotarget.

[25] Pishas KI, Neuhaus SJ, Clayer MT, Schreiber AW, Lawrence DM, Perugini M (2014). Nutlin-3a efficacy in sarcoma predicted by transcriptomic and epigenetic profiling. Cancer Res.

[26] Kubota D, Mukaihara K, Yoshida A, Suehara Y, Saito T, Okubo T (2013). The prognostic value of pfetin: a validation study in gastrointestinal stromal tumors using a commercially available antibody. Jpn J Clin Oncol.

